# Atopic dermatitis-alleviating effects of *lactiplantibacillus plantarum* LRCC5195 paraprobiotics through microbiome modulation and safety assessment via genomic characterization and in vitro analysis

**DOI:** 10.1038/s41598-025-16102-5

**Published:** 2025-08-20

**Authors:** Ahyoung Lim, Jihye Baek, Minju Seo, Ki-young Kim, Seonghan Kim, Woongkwon Kwak, Jaeho Lee, Jungki Kwak, Won-Joo Yoon, Wonyong Kim, Seokmin Yoon

**Affiliations:** 1 Lotte R&D Center, Seoul, 07594 Republic of Korea; 2 Department of Microbiology, Chung-Ang University College of Medicine, Seoul, 06974 Republic of Korea; 3 LuxBiome Co. Ltd, Seoul, 06974 Republic of Korea

**Keywords:** Atopic dermatitis, *Lactiplantibacillus plantarum*, Paraprobiotics, Gut microbiome, Genome characterization, Safety, Applied microbiology, Microbiome

## Abstract

The efficacy of paraprobiotics from *Lactiplantibacillus plantarum* LRCC5195 (LP5195-P) in alleviating atopic dermatitis (AD) through microbiome modulation and its safety were evaluated in AD-induced mice. Oral administration of LP5195-P to mice for 8 weeks after AD induction was used to investigate changes in microbiota, immune regulation, and symptoms of dermatitis. Taxonomic analysis of the gut microbiota revealed substantially higher bacterial diversity and abundance in LP5195-P treated group compared to that in negative control. Metabolic analysis revealed significant changes in short-chain fatty acid levels. These microbiome changes correlated with alterations in immune modulation. Furthermore, LP5195-P treatment decreased gene expression related to Treg and Th2 responses in the ileum and skin. Improvements in AD symptoms, including edema and erythema, were observed, and inhibitory effects on histamine release and β-hexosaminidase activity were demonstrated. In conclusion, LP5195-P administration induced a balanced immune response involving gut microbiota and short-chain fatty acids, highlighting its potential as a therapeutic candidate for AD.

## Introduction

Atopic dermatitis (AD) is a common, chronic skin disorder characterized by skin barrier dysfunction, inflammation, and immune dysregulation^1,2^. It predominantly affects infants and young children, with 60% of cases occurring within the first year of life and up to 85% by age 5. However, AD also persists into adulthood, with increasing prevalence over time^3,4^. The causes of AD have not been clearly defined, and current treatments, including environmental modifications, steroids, moisturizers, antihistamines, and antibiotics, are used to manage symptoms. However, the long-term use of steroids and anti-histamines is associated with the risk of resistance and adverse effects, emphasizing the need to limit their long-term use^5,6^. Consequently, an increasing number of studies have focused on safer alternatives to current treatments that effectively alleviate AD symptoms while mitigating the risk of adverse effects.

Numerous studies have reported a relationship between AD and the intestinal microflora. The term ‘microbiome’ refers to the entire genome of inhabiting microbes or microbiota, whereas the ‘human microbiome’ refers to the microbiome within an individual human body. The microbiome consists of over 200 times more genes than the human genome and is referred to as the “second genome”^7–9^. Previous studies have explored the association between the gut microbiota and specific diseases, including obesity, diabetes, depression, and AD, indicating that the microbiome has potential advantages in the prevention and management of various diseases^10–15^.

Probiotics are beneficial microorganisms that exert health effects on the host and are known for their role in microbiome regulation. Cell components and metabolites derived from these probiotics have been termed postbiotics and their beneficial effects have been reported^16,17^. In contrast to probiotics, postbiotics are not affected by gastric or bile acids and can be delivered to the intestines to exert their effects. In addition, their high stability under extreme conditions, such as high temperature or pressure, allows applicability across various fields. Similarly, paraprobiotics, which refer to inactivated (non-viable) microbial cells, also exhibit high stability and have been shown to exert beneficial effects on the host. In particular, they can modulate immune responses by suppressing Th2-dominant inflammation, as indicated by decrease in IgE levels and Th2 cytokine expression^18^. Consequently, there has been growing interest in the use of postbiotics as alternatives to probiotics for the treatment of AD. Choi et al. reported that treatment with heat-killed *Enterococcus faecalis* EF-2001 significantly decreased IgE, IL-4, IL-5, and IL-22 levels^19^. Furthermore, Kim et al. reported that the administration of cream cheese-derived *Lactococcus chungangensis* CAU 28 regulated the microbiome and AD-related inflammatory cytokines in animals^8^. Although probiotics are widely recognized for their health benefits and are generally considered safe, adverse effects have been reported. Although these side effects are relatively rare compared to those of other pharmaceuticals or natural products^20,21^, concerns regarding their safety persist. Accordingly, many countries have established guidelines for evaluating the safety of probiotics, with antibiotic susceptibility, cytotoxicity, toxic metabolites, and hemolytic activity recommended as the key assessment criteria. Traditionally, the evaluation relies primarily on in vitro assays. However, recent advancements in genomic analysis technologies have led to more precise assessments, including genomic characterization.

This study analyzed changes in gut microbiome diversity and abundance following the administration of paraprobiotics derived from *Lactiplantibacillus plantarum* LRCC5195 (LP5195) in an AD-induced mouse model (4 groups, *n* = 6 per group). Furthermore, relationships among microbiota modulation, immune regulation, and alleviation of dermatitis symptoms were investigated. Additionally, safety assessments including genomic characterization and in vitro analysis were conducted to evaluate the safety of the strain.

## Results

### Effects on AD symptoms and on mRNA expression related to inflammation

**Fig. 1:**
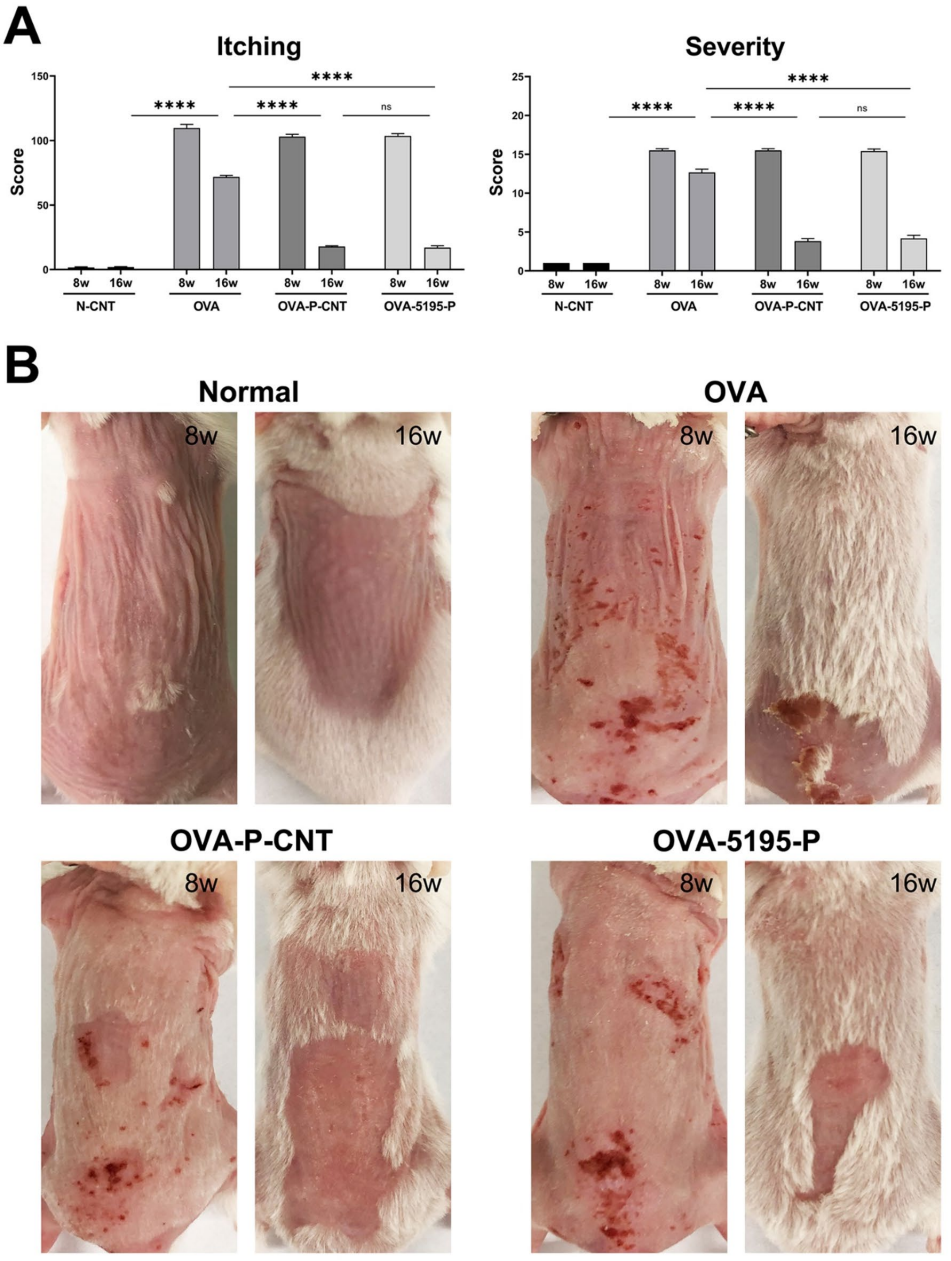
Assessment of atopic dermatitis symptoms with *Lactiplantibacillus plantarum* LRCC5195 paraprobiotics treatment. (**A**) Itching and severity scores. (**B**) Gross examination of skin lesions at weeks 8 and 16. OVA: AD-induced animal groups with ovalbumin; OVA-P-CNT: AD-induced animal groups with ovalbumin and commercial ‘*L. plantarum* product C’; OVA-5195-P: AD-induced animal groups with ovalbumin and *Lactiplantibacillus plantarum* LRCC5195 paraprobiotics. Statistical significance between groups is indicated by underlined text and asterisks: *P* < 0.05 (*), *P* < 0.01 (**), *P* < 0.001 (***), *P* < 0.0001 (****). ^ns^Means no significant differences between the groups.

Ovalbumin treatment induced distinct AD lesions, including dryness, excoriation, edema, erosion, and erythema, with significant differences between the OVA-P-CNT (animals with AD induction and treated with a commercially recognized probiotic strain) and OVA-5195-P groups (animals with AD induction and treated with LP5195-P; LP5195-P: LP5195-derived paraprobiotics) (Fig. 1A). After AD induction at week 8, both itching and severity scores notable decreased at week 16 compared to those at week 8. Additionally, at week 16, both the OVA-P-CNT (*P* < 0.0001, both) and OVA-5195-P groups (*P* < 0.0001, both) exhibited significantly lower itching and severity scores than the OVA (animals with AD induction and treated with PBS) group. Gross examinations at weeks 8 and 16 revealed notable differences between the groups (Fig. 1B). Skin lesions persisted in the OVA group at week 16, whereas most lesions showed a marked improvement in the OVA-P-CNT and OVA-5195-P groups.

**Fig. 2:**
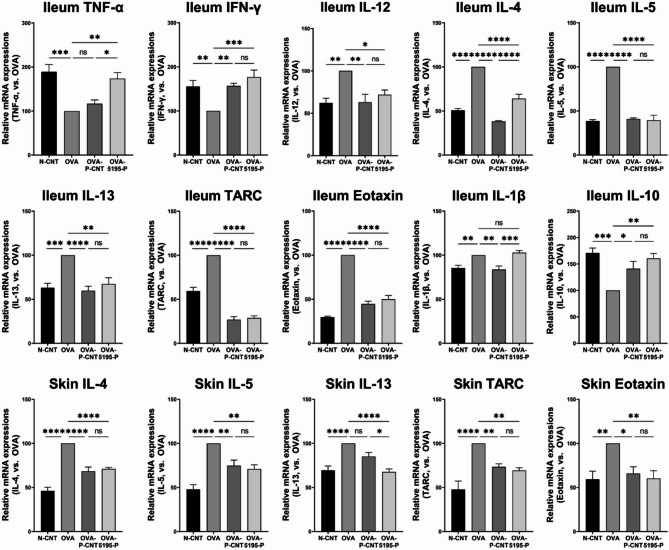
Effects of *Lactiplantibacillus plantarum* LRCC5195 paraprobiotics on mRNA expression related to inflammation. N-CNT: animal groups fed a normal diet; OVA: AD-induced animal groups with ovalbumin; OVA-P-CNT: AD-induced animal groups with ovalbumin with commercial ‘*L. plantarum* product C’; OVA-5195-P: AD-induced animal groups with ovalbumin with *Lactiplantibacillus plantarum* LRCC5195 paraprobiotics. Ileum: Analyzed mRNA expression levels of cytokines from ileum tissues; Skin: Analyzed mRNA expression levels of cytokines from skin tissues. Statistical significance between groups is indicated by underlined text and asterisks: *P* < 0.05 (*), *P* < 0.01 (**), *P* < 0.001 (***), *P* < 0.0001 (****). ^ns^Means no significant differences between the groups.

The mRNA expression levels of inflammation-related cytokines in the ileum and skin tissues were analyzed (Fig. 2). In the ileum, the mRNA expression levels of IL-12, IL-4, IL-5, IL-13, TARC, and eotaxin were significantly lower in the OVA-P-CNT (*P* = 0.0020, *P* < 0.0001, *P* < 0.0001, *P* < 0.0001, *P* < 0.0001, and *P* < 0.0001, respectively) and OVA-5195-P groups (*P* = 0.0191, *P* < 0.0001, *P* < 0.0001, *P* = 0.0010, *P* < 0.0001, and *P* < 0.0001, respectively) than in the OVA group. In contrast, IFN-γ expression was significantly higher in both the OVA-P-CNT and OVA-5195-P groups (*P* = 0.0077, and *P* = 0.0004, respectively), while TNF-α was significantly increased only in the OVA-5195-P group (*P* = 0.0010). Additionally, the levels of IL-10, an anti-inflammatory cytokine, were significantly higher in both the OVA-P-CNT and OVA-5195-P groups than in the OVA group (*P* = 0.0284, and *P* = 0.0011, respectively). IL-1β mRNA expression was significantly lower in the OVA-P-CNT group compared to the OVA group (*P* = 0.0027), while no significant difference was observed in the OVA-5195-P group (*P* = 0.8906). In the skin tissues, IL-4, IL-5, TARC, and eotaxin mRNA expression levels were significantly lower in both the OVA-P-CNT (*P* < 0.0001, *P* = 0.0072, *P* = 0.0100, and *P* = 0.0188, respectively) and OVA-5195-P groups (*P* < 0.0001, *P* = 0.0020, *P* = 0.0029, and *P* = 0.0057, respectively) than in the OVA group. IL-13 expression was significantly lower in the OVA-5195-P group (*P* < 0.0001).

### Effects on serum cytokines, and histological changes

**Fig. 3:**
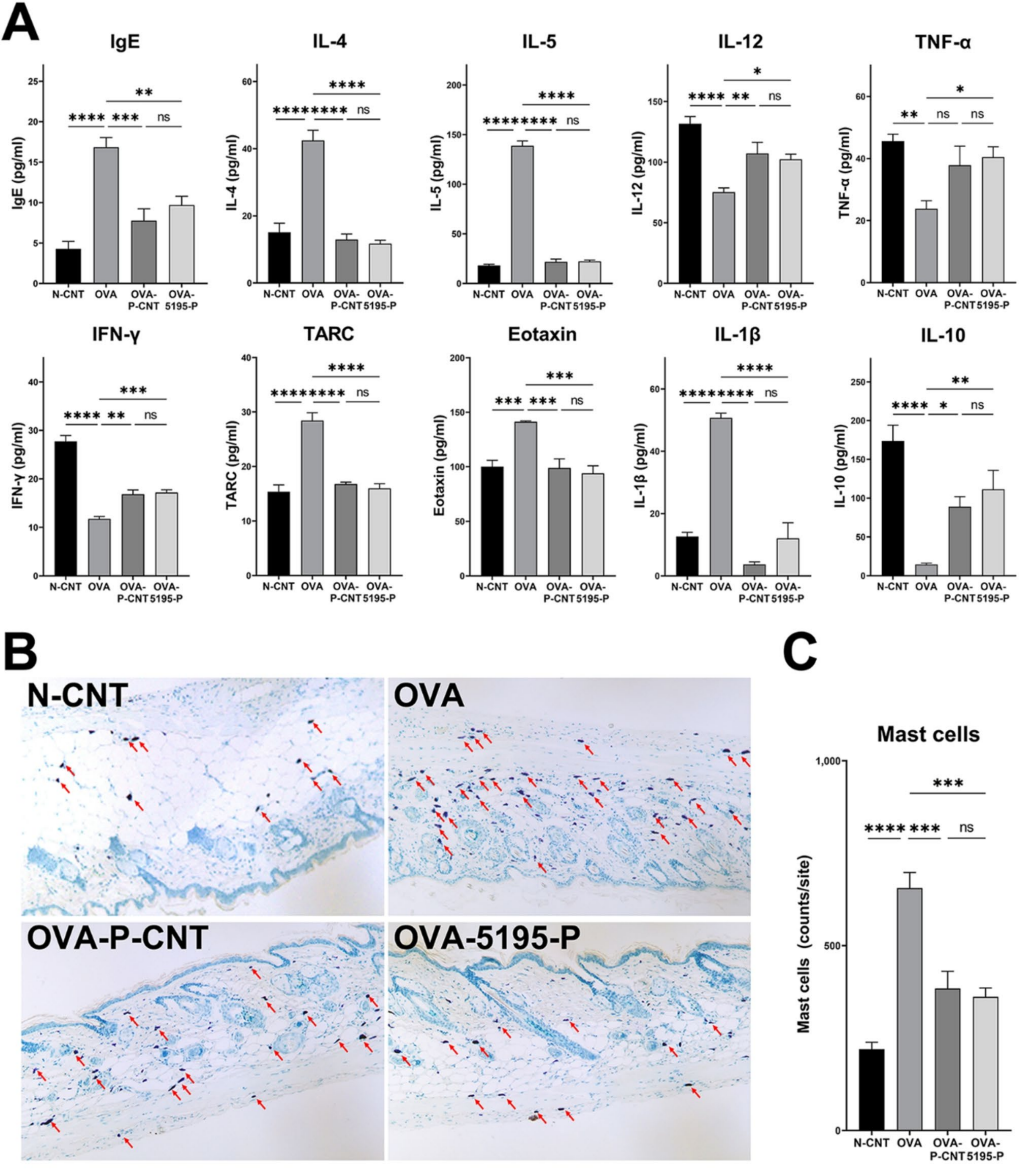
Cytokine levels and histological analysis with *Lactiplantibacillus plantarum* LRCC5195 postbiotics treatment. (**A**) Cytokine levels in serum. (**B**) H&E staining of skin tissue. (**C**) Quantification of mast cells. N-CNT: animal groups fed a normal diet; OVA: AD-induced animal groups with ovalbumin; OVA-P-CNT: AD-induced animal groups with ovalbumin with commercial ‘*L. plantarum* product C’; OVA-5195-P: AD-induced animal groups with ovalbumin with *Lactiplantibacillus plantarum* LRCC5195 paraprobiotics. Statistical significance between groups is indicated by underlined text and asterisks: *P* < 0.05 (*), *P* < 0.01 (**), *P* < 0.001 (***), *P* < 0.0001 (****). ^ns^Means no significant differences between the groups.

The results of serum cytokine analysis are shown in Fig. 3A. The IgE levels were significantly lower in the OVA-P-CNT and OVA-5195-P groups than in the OVA group (*P* = 0.0001, and *P* = 0.0020, respectively). The levels of Th2 cytokines, including IL-4, IL-5, TARC, and eotaxin were significantly lower in the OVA-P-CNT (*P* < 0.0001, *P* < 0.0001, *P* < 0.0001, and *P* = 0.0005, respectively), and OVA-5195-P groups (*P* < 0.0001, *P* < 0.0001, *P* < 0.0001, and *P* = 0.0001, respectively) than in the OVA group. Additionally, IL-1β levels were significantly lower in the OVA-P-CNT and OVA-5195-P groups than in the OVA group (*P* < 0.0001, *P* < 0.0001, *P* < 0.0001, and *P* = 0.0001, respectively). In contrast, the levels of IL-12, and IFN-γ were significantly higher in the OVA-P-CNT and OVA-5195-P groups compared to the OVA group (*P* < 0.0001, both). TNF-α levels were significantly higher only in the N-CNT (animals without AD induction and without any treatment) and OVA-5195-P groups compared to the OVA group (*P* = 0.0039, and *P* = 0.0308, respectively). The levels of IL-10, an anti-inflammatory cytokine, were significantly higher in the OVA-P-CNT and OVA-5195-P groups than in the OVA group (*P* = 0.0281, and *P* = 0.0036, respectively). Significant differences were observed in the levels of all cytokines between the N-CNT and OVA groups.

The H&E staining results of skin tissues from the sacrificed animals are shown in Fig. 3B and C. Histological examination revealed the highest number of infiltrative mast cells (red arrow) in the OVA group, whereas a decrease was observed in both OVA-P-CNT and OVA-5195-P groups. Quantitative analysis consistently demonstrated that the number of mast cells was significantly lower in the OVA-P-CNT and OVA-5195-P groups than that in the OVA group (*P* = 0.0007 and *P* = 0.0003, respectively).

## Modulation of gut microbiota

**Fig. 4:**
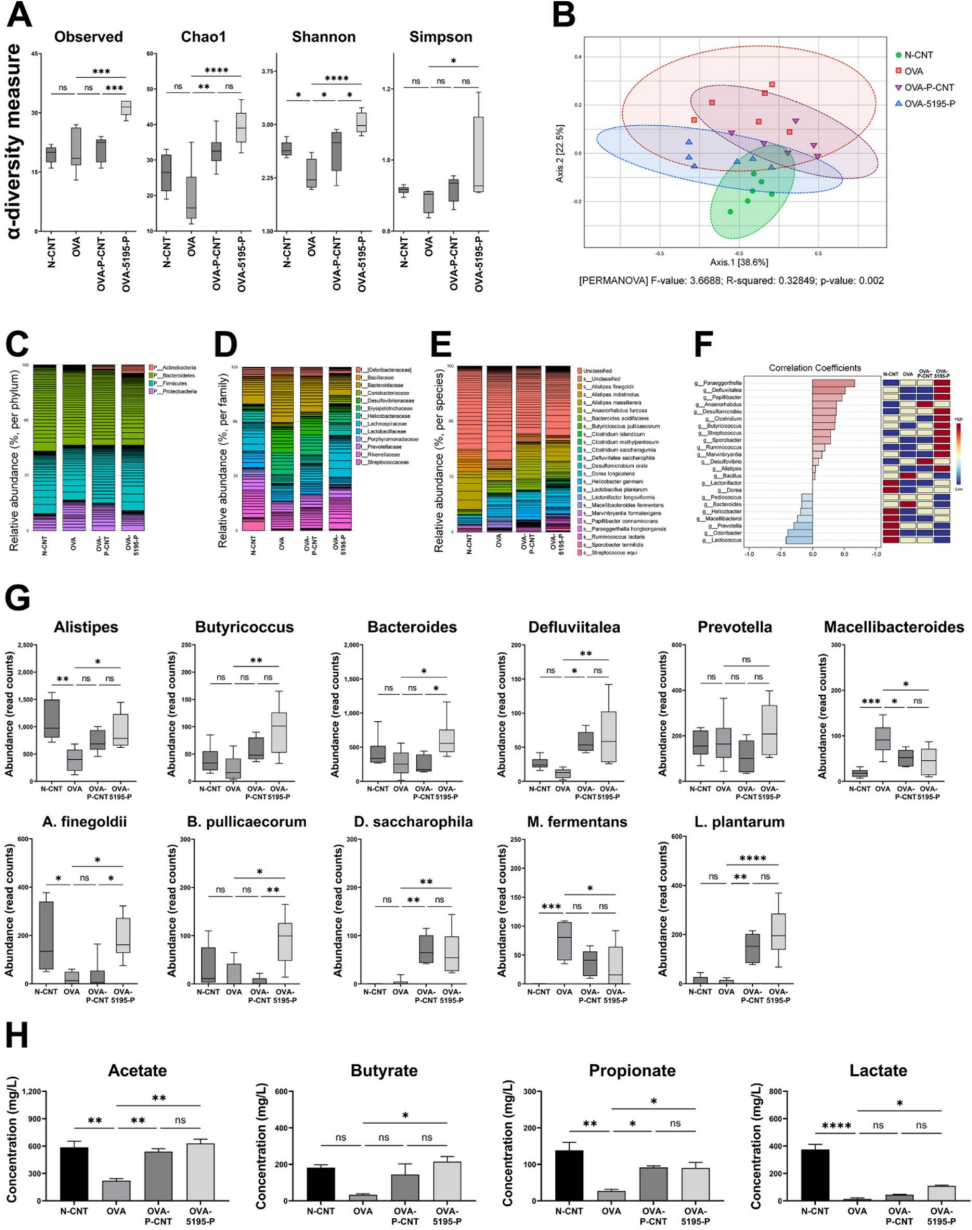
Modulation of gut microbiota by *Lactiplantibacillus plantarum* LRCC5195 paraprobiotics. (**A**) α-diversity indices (Observed species, Chao1, Shannon, and Simpson). (**B**) β-diversity analysis. (**C**) Relative abundance at the phylum level. (**D**) Relative abundance at the family level. (**E**) Relative abundance at the phylum level. (**F**) genus level heatmap hierarchical clustering. (**G**) Relative abundance of genera. (**H**) Short-chain fatty acids and lactic acid. N-CNT: animal groups fed normal diet; OVA: AD-induced animal groups fed OVA. OVA-P-CNT: AD-induced animal groups with ovalbumin with commercial ‘*L. plantarum product* C’; OVA-5195-P: AD-induced animal groups with ovalbumin with *Lactiplantibacillus plantarum* LRCC5195 paraprobiotics. Statistical significance between groups is indicated by underlined text and asterisks: *P* < 0.05 (*), *P* < 0.01 (**), *P* < 0.001 (***), *P* < 0.0001 (****). ^ns^Means no significant differences between the groups.

The following four experimental groups, each consisting of six mice, were assigned to assess the effects of LP5195-derived paraprobiotics (LP5195-P) on gut microbiota: N-CNT (animals without AD induction and without any treatment), OVA (animals with AD induction and treated with PBS), OVA-5195-P (animals with AD induction and treated with LP5195-P), and OVA-P-CNT (animals with AD induction and treated with a commercially recognized probiotic strain). The indices of α-diversity and β-diversity for the animals are shown in Fig. 4A and B. The number of observed species and Chao1, Shannon, and Simpson indices in the OVA-5195-P group (AD-induced with ovalbumin, and treated with paraprobiotics of LP5195) were significantly different from those in the OVA group (*P* = 0.0001, *P* < 0.0001, *P* < 0.0001, and *P* = 0.0478, respectively). Overall, the OVA-5195-P group exhibited the greatest diversity and evenness in gut microbiota. In the OVA-P-CNT group, the α-diversity indices were also higher than those in the OVA group, with significant differences observed in the Chao1 and Shannon indices (*P* = 0.0061, *P* = 0.0364, respectively). However, the observed species number and Simpson’s index showed no significant differences (*P* = 0.9746 and *P* = 0.8261, respectively). β-diversity analysis was conducted to assess the differences in microbial community profiles among the normal control, OVA, OVA-P-CNT, and OVA-5195-P groups. A significant difference was observed between the OVA and OVA-5195-P groups (*P* = 0.002). Relative abundance was analyzed to determine the composition of intestinal microbes in the experimental groups. Microbial composition was analyzed and visualized at the phylum, family, and species levels (Fig. 4C and E). For each level, taxa with a relative abundance below 1% were excluded from the visualization. The subdivision across taxonomic levels (e.g., from phylum to family) reflects the hierarchical structure of microbial taxonomy and was intentionally applied to illustrate compositional differences at multiple resolutions. Figure 4F shows the correlation coefficients and heatmap clustering results at the genus level for the animal groups. The analysis results revealed that the genera with the highest correlation coefficients were *Paraeggerthella*, *Defluviitalea*, *Papillibacter*, and *Desulfomicrobil* whereas *Lactococcus*, *Odoribacter*, and *Prevotella* exhibited the lowest correlation coefficients.

The relative abundances of the identified genera and species are shown in Fig. 4G. The genera that exhibited a significantly higher abundance in the OVA-5195-P group than in the OVA group included *Alistipes*, *Butyricicoccus*, *Bacteroides*, and *Defluviitalea* (*P* = 0.0239, *P* = 0.0030, *P* = 0.0482, and *P* = 0.0043, respectively). In contrast, *Macellibacteroides* abundance was significantly lower in the OVA-5195-P group than in the OVA group (*P* = 0.0163). No significant differences were observed in the abundance of *Prevotella* (*P* = 0.7778). In the OVA-P-CNT group, the genera that exhibited significant differences in abundance compared to the OVA group were *Defluviitalea* and *Macellibacteroides* (*P* = 0.0191, and *P* = 0.0458, respectively). No significant differences were observed for the other genera. Furthermore, significant differences were found between the OVA and N-CNT groups in the abundance of *Alistipes* and *Macellibacteroides* (*P* = 0.0019, and *P* = 0.002, respectively), whereas no significant differences were observed for the other genera. The species that exhibited a significantly higher abundance in the OVA-5195-P group than in the OVA group were *Alistipes finegoldii*, *Butyricicoccus pullicaecorum*, and *Defluviitalea saccharophila* (*P* = 0.0021, *P* = 0.0104, and *P* = 0.0052, respectively). In contrast, *Macellibacteroides fermentans* abundance was significantly lower in the OVA-5195-P group than in the OVA group (*P* = 0.0315). In the OVA-P-CNT group, only *Defluviitalea saccharophila* showed a significantly lower abundance compared to the OVA group (*P* = 0.002). The abundance of *Lactiplantibacillus plantarum* was significantly higher in both the OVA-5195-P and OVA-P-CNT groups compared to the OVA group (*P* = 0.0025 and *P* < 0.0001, respectively).

The concentrations of secreted SCFAs (acetic, propionic, and butyric acids) and lactic acid in the feces are shown in Fig. 4H. Concentrations of acetic, butyric, and propionic acids were significantly higher in the OVA-5195-P group than in the OVA group (*P* = 0.0011, *P* = 0.0211, and *P* = 0.0495, respectively). Additionally, the lactic acid concentration was higher in the OVA-5195-P group than in the OVA group (*P* = 0.0363). In the OVA-P-CNT group, the concentrations of acetic and propionic acids were significantly higher than those in the OVA group (*P* = 0.0054, and *P* = 0.0444, respectively), whereas no significant differences were observed for butyric and lactic acids (*P* = 0.1657, and *P* = 0.6953, respectively). In the OVA-P-CNTs and OVA-5195-P groups, the concentrations of acetic, butyric, and propionic acids were not significantly different from those in the N-CNTs group.

## Genomic characterization and annotation related to safety

The genome of LP5195 was 3,291,373 bp in length, with a GC content of 43.78%. The strain contains 3,213 coding sequences (CDS) along with 16 rRNA and 68 tRNA genes. The genomic map of LP5195 generated using the CGView tool is shown in Fig. 6. The gene annotation results based on WGS analysis of LP5195 are summarized in Table 1. LP5195 was deficient in most key genes associated with antibiotic resistance. Notably, the strain did not harbor bla_TEM, bla_SHV, vanA, or vanB, which are typically associated with resistance to beta-lactams and vancomycin. Additionally, aac(3)-II and ant(2’’)-Ia, which confer resistance to aminoglycosides, were absent. Similarly, the strA and strB genes, which are linked to streptomycin resistance, were not detected. In contrast, the presence of ermB suggests potential macrolide resistance, whereas cat confers chloramphenicol resistance, and msrA supports macrolide efflux pump activity, further indicating macrolide resistance mechanisms. Concerning hemolytic activity, the strain did not harbor genes typically associated with hemolysin production, including hlyA, hlyB, hlyC, and cylA, which are involved in beta-hemolysis and cytolysis, respectively. In addition, the plc gene, encoding phospholipase C, which is linked to alpha hemolysis, was not detected. This strain lacks genes related to cytotoxicity and lactate production. Although ldhA, associated with d-lactate dehydrogenase A activity, was present, most other genes involved in cytotoxic activity were deficient. Furthermore, ldhD, which is involved in d-lactate production, is deficient.

**Table 1:** Gene annotation of *Lactiplantibacillus plantarum* LRCC 5195 related to antibiotic susceptibility, hemolytic activity, cytotoxicity, and D-lactate production**.**

	Gene	EC No.	Product	LP5195
Antibiotic susceptibility	bla_TEM	3.5.2.6	Beta-lactamase TEM	-
	bla_SHV	3.5.2.6	Beta-lactamase SHV	-
	vanA	2.7.1.68	D-alanine-D-alanine ligase A	-
	vanB	2.7.1.68	D-alanine-D-alanine ligase B	-
	aac(3)-II	2.7.1.95	Aminoglycoside N-acetyltransferase	-
	ant(2’’)-Ia	2.7.7.47	Aminoglycoside O-nucleotidyltransferase	-
	strA	-	Streptomycin phosphotransferase A	-
	strB		Streptomycin phosphotransferase B	-
	ermB	2.1.1.184	Erythromycin resistance methylase B	+
	mefA	-	Macrolide efflux pump Mef(A)	-
	tetM	-	Ribosomal protection protein Tet(M)	-
	tetX	1.14.13.1	Tetracycline-modifying monooxygenase	-
	cat	2.3.1.28	Chloramphenicol acetyltransferase	+
	floR	-	Florfenicol/chloramphenicol efflux transporter	-
	msrA	-	Macrolide efflux pump Msr(A)	+
Hemolytic activity	hlyA	3.1.1.5	Hemolysin A (β-hemolysis)	-
	hlyB	-	Hemolysin secretion protein B (β-hemolysis)	-
	hlyC	-	Hemolysin secretion protein C (β-hemolysis)	-
	cylA	-	Cytolysis A (β-hemolysis)	-
	plc	3.1.4.3	Phospholipase C (α-hemolysis)	-
Cytotoxicity LDH release	Bax	-	Bcl-2-associated X protein	-
	caspase-3	3.4.22.56	Caspase-3 enzyme (executioner in apoptosis)	-
	ldhA	1.1.1.27	D-lactate dehydrogenase A	+
	ldhB	1.1.1.27	D-lactate dehydrogenase B	-
	ldhC	1.1.1.27	D-lactate dehydrogenase C	-
D-lactate production	ldhA	1.1.1.28	D-lactate dehydrogenase A	+
	ldhD	1.1.1.28	D-lactate dehydrogenase D	-

+: Gene detected in the genome; -: Gene not detected in the genome. EC No.: Enzyme commission number assigned to the gene product. Product: Functional product or enzyme encoded by the gene. LP5195: *Lactiplantibacillus plantarum* LRCC 5195.

### Safety assessments in vitro

The in vitro safety assessment of LP5195 is summarized in Table 2. Specifically, the MICs of LP5195 for ampicillin, gentamicin, erythromycin, clindamycin, tetracycline, and chloramphenicol were 0.125, 8, 0.5, 0.06, 8, and 1 µg/mL, respectively, all of which are within the acceptable ranges defined by EFSA standards. The EFSA standards do not require measurements for streptomycin, vancomycin, and tylosin, indicated as n.r.; however, testing revealed inhibition at concentrations of 0.5, 1, and 0.5 µg/mL for each antibiotic, respectively. In hemolytic activity, LP5195 did not exhibit α-, β-, or γ-hemolysis, indicating the absence of hemolysin production. In cytotoxicity assays, no significant changes in cell viability were observed at bacterial concentrations ranging from 10^7^ to 10^9^ CFU, with cell viability maintained between 96.2 ± 4.5% and 101.2 ± 3.6% compared to the untreated group. Lactate dehydrogenase release, a marker of cytotoxicity, ranged from 0.5 to 0.8%, indicating that the strain did not induce significant cytotoxic effects. Moreover, LP5195 produced 0.04 ± 0.1 g/L of D-lactate, indicating a limited capacity for D-lactate production. In the heat inactivation assessment, colony formation was observed after treatment at 70 °C for 20 and 30 min, and at 80 °C for 10 min. No colonies were detected following treatment at 80 °C for 20 and 30 min or at 90 °C for any tested duration, indicating that complete inactivation occurred under these conditions.

**Table 2:** Safety assessment of *Lactiplantibacillus plantarum* LRCC 5195 in vitro.

Antibiotic susceptibility					
Antibiotics	MIC (µg/mL)				
	EFSA standards			LP5195	
Ampicillin	2			0.125	
Vancomycin	n.r.*			0.2	
Gentamicin	16			8	
Kanamycin	64			64	
Streptomycin	n.r.			1	
Erythromycin	1			0.5	
Clindamycin	2			0.06	
Tetracycline	32			8	
Chloramphenicol	8			1	
Tylosin	n.r.			0.5	
**Hemolytic activity**					
Hemolysis types	LP5195				
α-hemolysis	-				
β-hemolysis	-				
γ-hemolysis	-				
**Cytotoxicity**					
	No treated/ Triton X-100			LP5195	
		10^7^ CFU		10^8^ CFU	10^9^ CFU
Cell viability (%)	101.5 ± 2.6	98.3 ± 5.1		101.2 ± 3.6	96.2 ± 4.5
LDH release (%)	104.4 ± 5.3	0.0 ± 0.2		0.1 ± 0.1	0.0 ± 0.2
**Lactate production**					
D-lactate (g/L)	0.04 ± 0.1				
L-lactate (g/L)	8.32 ± 0.2				
**Heat-inactivation**					
Temperature (℃)	Time (minutes)		Colony formation on MRS agar after streaking		
Initial	-		○		
70	20		○		
	30		○		
80	10		○		
	20		-		
	30		-		
90	10		-		
	20		-		
	30		-		

MIC: minimum inhibitory concentration for each antibiotic against *Lactiplantibacillus plantarum* LRCC 5195;. n.r.: not required or tested according to EFSA standards; ‘n.r.’ indicated “not required”;. EFSA standards: European Food Safety Authority standards;. LP5195: *Lactiplantibacillus plantarum* LRCC 5195;. 10^7^~10^9^ CFU: treated concentration of *Lactiplantibacillus plantarum* LRCC 5195. ‘○’ indicates the presence of colony growth; ‘–’ indicates the absence of colony formation.

### Effects of LRCC5195-P on immune modulation in vitro

**Fig. 5:**
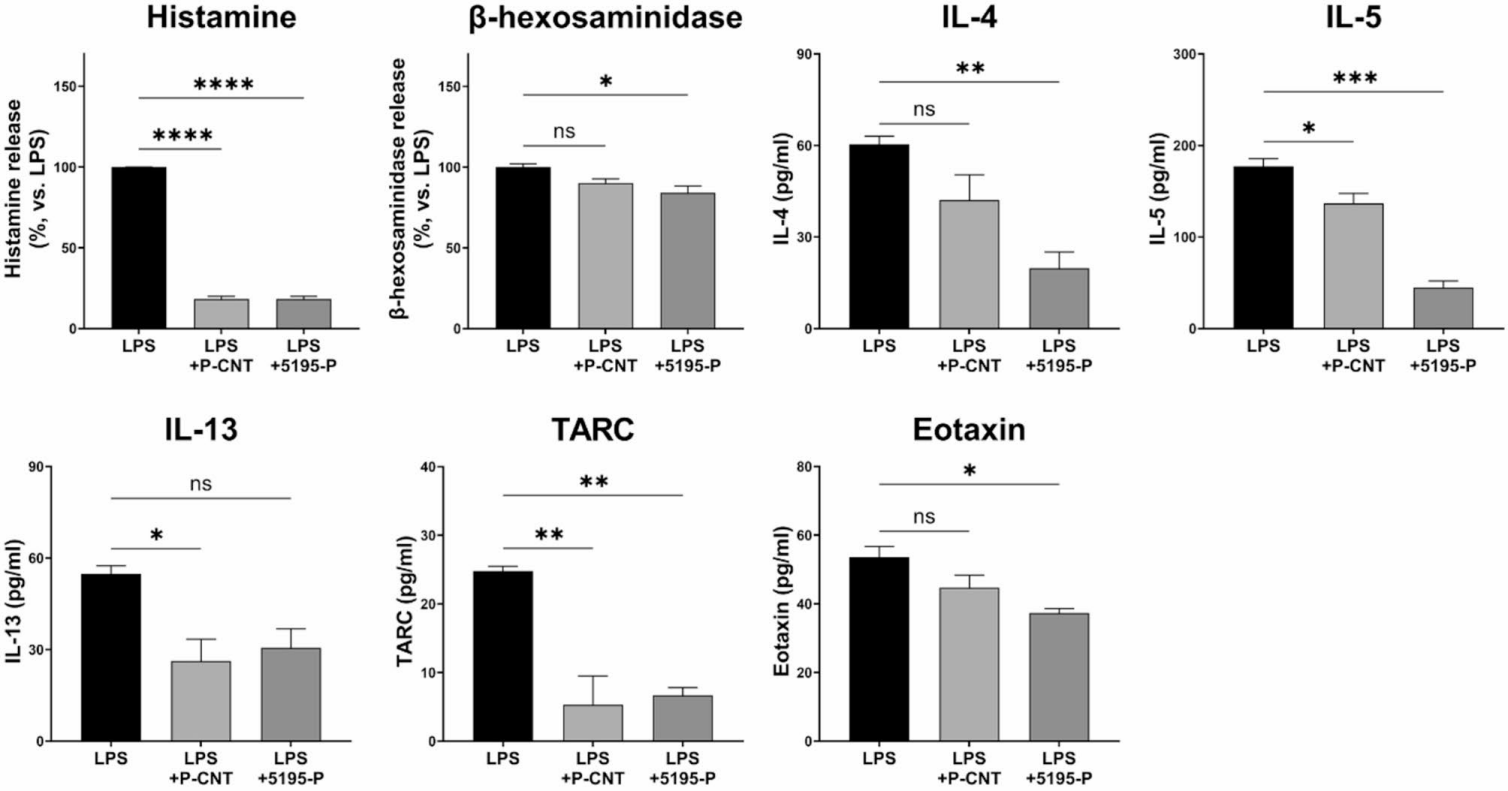
Effects of *Lactiplantibacillus plantarum* LRCC5195 paraprobiotics on immune modulation in vitro. LPS: lipopolysaccharide-treated group; LPS + P-CNT: treated with LPS and commercial ‘*L. plantarum* product C’; LPS + 5195-P: treated with LPS and *Lactiplantibacillus plantarum* LRCC5195 postbiotics. Statistical significance between groups is indicated by underlined text and asterisks: *P* < 0.05 (*), *P* < 0.01 (**), *P* < 0.001 (***), *P* < 0.0001 (****). ^ns^Means no significant differences between the groups.

Figure 5 shows the effects of LRCC5195-P treatment on RBL-2H3 and HaCaT cells. Both the OVA-P-CNT (*P* < 0.0001, *P* = 0.0035, and *P* = 0.0203, respectively) and OVA-5195-P (*P* < 0.0001, *P* = 0.0003, and *P* = 0.0006, respectively) groups exhibited significantly lower levels of histamine, TARC, and eotaxin than the LPS-treated group. In contrast, β-hexosaminidase, IL-4, IL-5, and IL-13 levels were significantly decreased only in the OVA-5195-P group (*P* = 0.0090, *P* = 0.0002, *P* < 0.0001, and *P* = 0.0051, respectively).

**Fig. 6:**
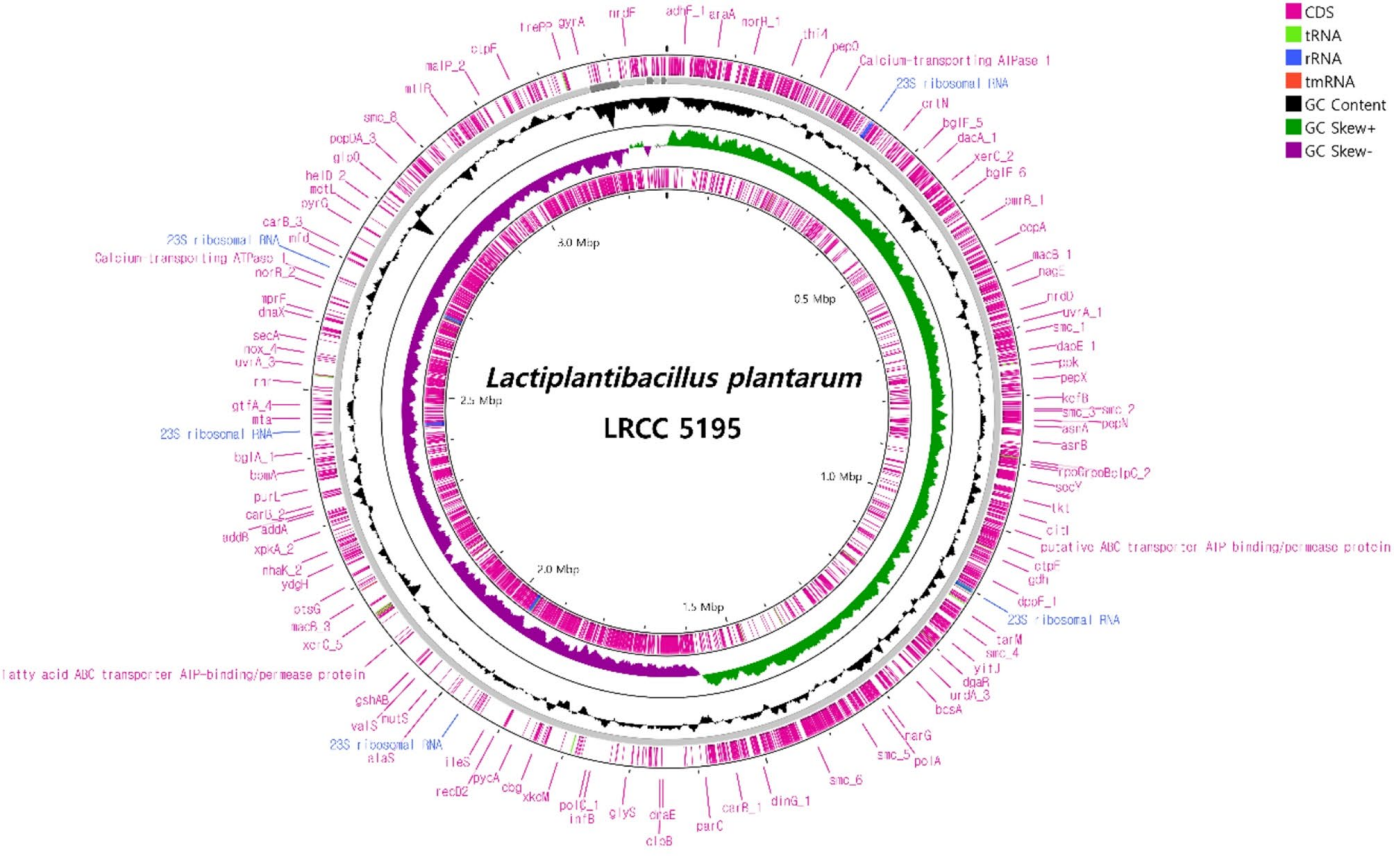
Circular map of the *Lactiplantibacillus plantarum* LRCC5195 genome visualized using the CGView tool. Genomic features are marked from the outer to the inner circle as follows: circles 1 and 2 illustrate Prokka-annotated forward and reverse coding sequences (CDS), respectively, with tRNA, rRNA, and tmRNA; circle 3 represents GC content; circle 4 shows the GC skew (G-C)/(G + C); and circle 5 shows genome size. GC, guanine cytosine.

## Discussion

Increasing evidence has indicated that gut microbiota dysbiosis is closely linked to various chronic diseases, including autoimmune disorders, allergies, metabolic syndromes, and particularly AD^9,22^. The modulation of the gut microbiota by probiotics, prebiotics, and postbiotics has been investigated as a potential strategy for managing AD.

AD is typically associated with severe skin symptoms including erythema and edema, which precipitate itching. These symptoms are mainly caused by immune imbalance, a well-known factor in the pathogenesis, as previously discussed. In a study by Park et al., the administration of *Lactobacillus helveticus* to AD-induced mice resulted in a significant decrease in AD symptoms, as indicated by evaluation using the CADESI scale^23^. Similarly, in another study, *Lactiplantibacillus plantarum* 22 A-3 administration led to a decrease in dermatitis symptoms, trans-epidermal water loss, and scratching behavior compared to those in the control group^24^. This immune imbalance triggers the production of inflammatory mediators that expand blood vessels, increase blood flow, and cause erythema. Increased vascular permeability allows fluid to leak into tissues, leading to edema. Consequently, an impaired skin barrier function results in increased water loss, dryness, and heightened sensitivity to external stimuli, further exacerbating inflammation and inducing itching. Notably, these improvements in AD symptoms have been suggested to result from enhanced immune regulation and increased diversity and abundance of the gut microbiota.

Although the exact cause of AD remains unclear, it is recognized as an immune hypersensitivity disorder, and immune modulation is a critical factor in its diagnosis, improvement, and treatment^7^. When an allergen enters the body, it binds to IgE on the surface of mast cells, leading to crosslinking with the Fc epsilon receptor (FcεR), which triggers the release of inflammatory mediators including histamine, serine proteinases, and serotonin, exacerbating the inflammatory response^25^. Additionally, the Th1/Th2 balance plays a crucial role in immune regulation. Th1 cytokines, including IFN-γ, TNF-α, and IL-12, promote cellular immunity by enhancing macrophage function, while Th2 cytokines, including IL-4 and IL-5, stimulate B cell antibody production and activate humoral immunity^26,27^. The Th1/Th2 balance is essential for the regulation of mast cell activation, and Th2-driven inflammation often leads to increased mast cell activity, which further exacerbates allergic responses. This increase in mast cells exacerbates the inflammatory response by secreting histamine, serine proteinases, carboxypeptidase A, and eicosanoid mediators^25^. IL-10, an anti-inflammatory cytokine, modulates this process by inhibiting the activation of pro-inflammatory immune cells, including mast cells, thus maintaining immune homeostasis. Therefore, modulating mast cell activity through cytokine regulation, particularly by promoting a favorable Th1/Th2 balance and enhancing IL-10 levels, represents a potential therapeutic strategy for mitigating AD symptoms. Treatment with 5195-P led to significant changes in cytokine levels compared to those in the OVA group. Specifically, the levels of IFN-γ, TNF-α, and IL-12 were elevated, while IL-4, and IL-5 levels decreased, aligning more closely with the control group. Additionally, the levels of TARC and eotaxin significantly decreased in the OVA-5195-P group, accompanied by a marked increase in IL-10, an anti-inflammatory cytokine. These cytokine alterations were further supported by histological and quantitative analyses that revealed a significant increase in the number of mast cells in the OVA group. The notable decrease in mast cell numbers in the 5195-P-treated group indicated that the adjustment of cytokine levels to those comparable to the control group contributed to this effect. Moreover, the mRNA expression of the corresponding cytokines in both the ileum and skin tissues was analyzed, and the expression levels were found to correlate with cytokine levels and mast cell numbers. Thus, the results showing the modulated immune responses with 5195-P treatment suggest its potential to improve AD-related symptoms and other immune-related diseases.

Microbial diversity is a key factor that influences immune regulation, and a decrease in gut microbiota diversity is frequently associated with inflammatory disorders, including AD. In other words, a more diverse microbiota is generally linked to improved immune stability against environmental stress. Several studies have reported that reduced microbiota diversity is associated with an increased risk of AD, whereas higher diversity in early life is associated with a lower incidence of AD^8,28^. These findings suggest that microbial diversity may play a role in the modulation of AD symptoms. In this study, α-diversity indices (observed species, Chao1, Shannon, and Simpson) were significantly higher in the OVA-5195-P group compared to the OVA group, suggesting that 5195-P administration contributed to a more diverse and stable gut microbiome. Furthermore, β-diversity analysis showed significant differences in the microbiome composition between the OVA-5195-P and OVA groups. These findings suggest that 5195-P administration increases microbial diversity and alters microbial composition, potentially contributing to immune modulation.

The relative abundance of specific microbial taxa is another key parameter in gut microbiome regulation because alterations in microbial composition are closely linked to immune homeostasis and inflammatory processes. In the present study, AD induction with OVA resulted in a decrease in *Firmicutes*, whereas *Proteobacteria* exhibited a marked increase at the phylum level. However, following 5195-P treatment, the proportions of *Firmicutes* and *Proteobacteria* were regulated and adjusted to levels comparable to those observed in the N-CNT group. Previous studies have shown to be associated with an imbalance in the relative abundance of *Bacteroidetes*, *Firmicutes*, and *Proteobacteria*^2,29^. *Bacteroidete*s and *Firmicutes* are involved in maintaining gut stability and immune regulation, and their increased abundances after 5195-P treatment may contribute to the regulation of AD-associated immune imbalances. In contrast, *Proteobacteria* are associated with proinflammatory responses, and their decreased abundance in the 5195-P group further supports their potential role in immune modulation. At the genus level, AD induction resulted in an increased abundance of *Macellibacteroides* and other genera associated with dysbiosis under inflammatory conditions. Notably, the abundance of *Alistipes* was significantly reduced in the OVA group, consistent with recent studies demonstrating that *Alistipes* contributes to gut immune homeostasis and exerts anti-inflammatory effects in murine models of colitis and dermatitis^30,31^. Additionally, the decreased abundance of *Butyricicoccus*, a genus known for producing short-chain fatty acids and regulating intestinal immunity, suggests decreased microbiota-derived anti-inflammatory activity^32,33^. Moreover, the decreased abundance of *Defluviitalea*, previously shown to be inversely associated with pro-inflammatory cytokines, notably TNF-α^34^, may reflect an altered microbial profile linked to inflammatory exacerbation. Although *Defluviitalea* is generally regarded as a thermophilic genus, its 16 S rRNA gene sequences have been detected in mammalian gut microbiome studies, including murine models of inflammation^35,36^. This may indicate the presence of strain-level variants potentially capable of adapting to mesophilic environments. Conversely, 5195-P treatment significantly increased the abundance of *Alistipes*, *Butyricicoccus*, *Bacteroides*, and *Defluviitalea*, while significantly decreasing *Macellibacteroides*, suggesting that these microbial changes may have contributed to the improvement of gut microbial balance.

While it may be inappropriate to attribute direct causality to species-level alterations in the pathogenesis of AD, such analyses remain valuable for a more integrated interpretation when evaluated alongside phylum- and genus-level microbial shifts. *Alistipes finegoldii* and *Butyricicoccus pullicaecorum* are of particular interest due to their reported roles in immunomodulation. *(A) finegoldii* has been characterized as a commensal bacterium frequently depleted in inflammatory conditions^37^, whereas *(B) pullicaecorum*, a butyrate-producing species^38^, has been linked to anti-inflammatory activity through enhancement of epithelial barrier integrity and regulation of immune responses. In addition, *Defluviitalea saccharophila*, while not previously reported in direct association with AD, exhibited an abundance pattern consistent with the *Defluviitalea* genus^34,39^, which has been inversely correlated with inflammatory conditions. This observation may imply a potential inverse relationship with immune dysregulation. Conversely, *Macellibacteroides fermentans*, a pathogenic species frequently increased in the oral microbiota of patients with chronic periodontitis^40^, has not been directly associated with AD. However, an increase in the *Macellibacteroides* genus has been reported under AD-related conditions, suggesting that the observed abundance of *M. fermentans* may be linked to inflammatory dysbiosis in AD. Furthermore, *L. plantarum* has been extensively investigated for its immunomodulatory capabilities, particularly its role in regulating the Th1/Th2 balance and enhancing epithelial barrier integrity^41^. In the present study, its relative abundance increased following administration of P-CNT and 5195-P, which is presumably due to the inclusion of *L. plantarum* in both preparations. Further research is necessary to determine whether this increase is indicative of active colonization or merely represents a short-term rise associated with oral administration. Considering the previously reported roles of the altered microbial composition, this microbial shift may be associated with a potential contribution to alleviating inflammation. Although the current study provides insight into the microbial modulation effects of 5195-P, the sample size (*n* = 6 per group), selected based on precedent from related animal studies, may limit the statistical power of microbiome analysis. To improve the robustness and generalizability of these findings, future studies should incorporate a larger number of animals per group.

Metabolites produced by gut microbiota play a critical role in host physiology and immune function^42,43^. SCFAs are key biomarkers for maintaining health and contributing to disease pathogenesis^8,9^. SCFAs function as energy sources, substrates, and signaling molecules and are closely involved in the metabolism of lipids, glucose, and cholesterol^44^. Notably, butyrate, a prominent SCFA, plays a pivotal role in regulating immune responses in macrophages and promoting Treg formation, and is produced by members of the *Lachnospiraceae* and *Ruminococcaceae* families^8,45^. In the OVA-induced model treated with 5195-P, an increase in the abundance of the *Lachnospiraceae* family, including *Defluviitalea* and *Marvinbryantia*, and the *Ruminococcaceae* family, including *Papillibacter* and *Sporobacter*, was observed. These changes suggest that the increase in these genera likely contributes to the elevation of SCFAs in the gut microbiome.

The genomic characterization and in vitro safety evaluation of LP5195 provided critical insights into its safety profile, supporting its potential as a probiotic. Notably, this strain lacked the key antibiotic resistance genes including bla_TEM, bla_SHV, tetM, tetX, aac(3)-II, strA, and strB. The genes, bla_TEM and bla_SHV, are associated with resistance to β-lactam antibiotics, including ampicillin and penicillin, enabling bacterial degradation of these antibiotics^42,46^. Additionally, tetM and tetX are associated with resistance to tetracycline and mediate this resistance by enzymatically modifying the antibiotic structure^47^. Therefore, the absence of these resistance genes suggests that LP5195 has low potential for acquiring antibiotic resistance and is unlikely to facilitate gene transfer to other bacteria. Additionally, genes associated with antibiotic resistance, such as ermB, cat, and msrA, were identified in the genomic analysis; however, no resistance was observed in the in vitro tests. This suggests that the absence of phenotypic resistance, despite the genomic presence of resistance-related genes such as ermB, cat, and msrA, may be attributed to incomplete open reading frames, mutations within regulatory elements, or the absence of functional promoters, all of which could impair the transcription and translation of active resistance proteins. Further studies are required to clarify the underlying genetic or regulatory mechanisms contributing to this inconsistency.

The safety of LP5195 was further evaluated using gene annotation and in vitro assays to assess its hemolytic activity, cytotoxicity, and D-lactate production. These factors are essential to evaluate the safety of probiotics. Hemolytic activity refers to the ability of microorganisms to lyse red blood cells, which can lead to tissue damage and anemia. Cytotoxicity involves the potential to damage host cells, causing inflammation and impairing immune function. Furthermore, excessive D-lactate production can lead to an imbalance in acid-base homeostasis, disrupting metabolic processes. The gene annotation results of LP5195 based on WGS analysis revealed that it did not harbor genes associated with hemolytic activity, including hlyA, hlyB, hlyC, cylA, and plc. Furthermore, the strain lacked most of the genes related to cytotoxicity and D-lactate production. Although ldhA was detected, as demonstrated by in vitro assays, no significant effects were observed. In vitro testing results showed that LP5195 demonstrated high cell viability (96.2 ± 4.5% to 101.2 ± 3.6%) across a range of bacterial concentrations (10^7^ to 10^9^ CFU), minimal LDH release (< 0.1%), and low D-lactate production (0.04 ± 0.1 g/L). Numerous studies evaluating the safety of probiotics have shown that many strains do not significantly affect cell viability when administered at concentrations of up to 10^8^ CFU. In addition, the release of lactate dehydrogenase, a marker of cytotoxicity, remained minimal and exhibited no significant variation across different strains within this concentration range^48,49^. Furthermore, Lee et al. reported that *Lacticaseibacillus* paracasei IDCC 3401 produced 0.41 g/L of D-lactate and 7.59 g/L of L-lactate, indicating low D-lactate production and suggesting the strain’s safety for human consumption^50^. In contrast, Lee et al. reported significantly higher L-lactate production, ranging from 14.33 to 28.12 g/L in four *Lactobacillus* strain^48^. This comparison underscores the relatively low D-lactate production by LP5195, further suggesting that it has minimal impact on metabolic disturbances. In addition, heat inactivation testing confirmed that LP5195 was no longer viable after treatment at 80 °C for at least 20 min or at 90 °C for any tested duration, suggesting that these conditions could be sufficient to achieve complete inactivation. This finding could support the safe use of heat-killed LP5195 as a paraprobiotic. Although the current study addressed several key safety parameters, a more comprehensive assessment would require additional evaluations. To comprehensively establish the safety profile of LP5195, future studies should include assays such as DNase activity, biogenic amine production, and translocation testing. These additional investigations would provide more definitive evidence supporting the strain’s suitability as a probiotic candidate.

In conclusion, LP5195-P exhibited significant immunomodulatory effects, including regulation of immune responses, cytokine levels, and mast cell activity, which contributed to the alleviation of AD symptoms. Evidence that these effects result from the increased diversity and balanced microbiome was provided, and safety was evaluated through WGS analysis and in vitro testing. However, this study has a limitation, particularly the use of animal models, which may not fully replicate the complexity of human AD pathology. Therefore, further research, including clinical trials in humans, is essential to confirm the efficacy and safety of LP5195-P in potential therapeutic applications. Moreover, while the safety results derived from the WGS analysis provided valuable insights, certain discrepancies were observed between the genomic data and in vitro findings. To improve the reliability of safety evaluations, it is essential to refine and enhance the accuracy of the WGS-based safety assessment tools in future studies.

## Materials and methods

### Isolation and postbiotic preparation

Lactic acid bacteria (LAB) were isolated from kimchi obtained from traditional Korean markets, diluted in sterile saline (0.85% NaCl), and plated onto de Man-Rogosa-Sharpe (MRS) agar medium (Difco) containing 0.02% bromocresol purple. After 48 h incubation at 37 °C, LAB were identified using 16 S rRNA gene sequencing. Amplification and sequencing were performed as previously described^39^, and the sequences were analyzed using the NCBI BLAST program (https://blast.ncbi.nlm.nih.gov/Blast.cgi). A total of 196 LAB strains were identified. Based on prior internal screening studies (data not shown in this study), which evaluated the ability of each isolate to modulate immune responses by enhancing IL-10 production and reducing IL-4 levels in vitro, LP5195 demonstrated the most prominent immunomodulatory profile. This finding led to its selection for further evaluation in the present study, and was the basis for its patent registration in Korea (Patent No. 10-2263698-0000). Paraprobiotics were prepared as described previously with some modifications^51^. LP5195 was inoculated into 10 mL of MRS broth in a 15 mL conical tube, and the tube was placed in a rectangular anaerobic jar (Rectangular Jar for AnaeroPack, Mitsubishi Gas Chemical Company, Tokyo, Japan) along with an AnaeroPack (AnaeroPack A-04, Mitsubishi Gas Chemical Company, Tokyo, Japan). The sealed jar was then incubated at 37 °C for 48 h in a 5% CO₂ incubator. After incubation, the culture was centrifuged (M1416R, Thermo Fisher Scientific, Waltham, MA, USA) at 8,000 × g for 10 min. The supernatant was removed, and the pellet was heat-treated at 90 °C for 30 min in a water bath (TSGP15D, Thermo Fisher Scientific). Prior to heat treatment, the viable cell count was adjusted to 1 × 10^9^ CFU/mL using a hemocytometer. To verify the accuracy of hemocytometer-based enumeration, viable cell counts were cross-validated by performing plate counts after 48 h of incubation. Following heat inactivation, the cell concentration was re-assessed using a hemocytometer, and the suspension was adjusted to 110–120% of the initial viable count. The resulting suspension was used as the postbiotic preparation from LP5195. For animal administration, the standardized postbiotic suspension (1 × 10⁹ cells/mL) was diluted with sterile saline to achieve a concentration of 1 × 10⁸ cells/200 µL, which was used as the treatment dose.

### AD-animal model

Five-week-old female BALB/c mice (Central Laboratory Animals, Seoul, Korea) were used after one week of adaptation. A total of 24 six-week-old female BALB/c mice were used in this study^2^. Body weights at the beginning of the experiment ranged from 20.5 to 23.4 g (22.1 ± 0.5 g). Animals were kept under controlled conditions: temperature 23 ± 1 °C, humidity 50 ± 5%, noise ≤ 60 dB, lighting from 06:00 to 18:00 (12 h daily), illumination 150–300 Lux, and ventilation 10–12 times per hour. Commercial ‘*L. plantarum* product C’ was used as a positive control (P-CNT). This strain has been recognized by the Ministry of Food and Drug Safety of Korea for its approved functionality in immune modulation and alleviation of AD. This strain was in probiotic form, and a commercially available product composed entirely of this strain, originally containing ≥ 1 × 10¹⁰ CFU/g, was purchased and used in the experiments without heat inactivation. To ensure dosage equivalence with LP5195-P, the product was diluted in sterile saline to a final concentration of 1 × 10⁸ CFU/200 µL, immediately prior to oral administration. The mice were randomly divided into four experimental groups (n = 6 per group)^2^: normal control (N-CNT), AD-induced^2,8^, treated with phosphate-buffered saline (OVA), AD-induced, treated with P-CNTs (OVA-P-CNT), and AD-induced, treated with LP5195-P (OVA-5195-P). AD was induced via intraperitoneal injection of ovalbumin (50 mg/mL, Sigma-Aldrich A5503, St. Louis, MO, USA) four times at 2-week intervals for 8 weeks^8,24^. Phosphate-buffered saline, P-CNT, and LP5195-P (10^8^ cells/animal, 200 µL) were administered once daily for 8 weeks following AD induction. After the treatment period, the animals were euthanized by exposure to 100% carbon dioxide CO₂ in a sealed chamber at a displacement rate of 30% of the chamber volume per minute^52,53^. Euthanasia was performed in accordance with the AVMA Guidelines for the Euthanasia of Animals to ensure gradual induction of unconsciousness while minimizing distress. Complete loss of consciousness and cessation of vital signs were confirmed prior to sample collection. All procedures were approved by the Animal Experimental Ethics Committee of Chung-Ang University (IACUC No. 2018-00022) and conducted in accordance with institutional ethical regulations and the ARRIVE guidelines (https://arriveguidelines.org).

### AD severity and mRNA expression of inflammation-related genes

The severity of AD was evaluated by recording skin symptoms before and after 8 weeks. To enable direct observation of skin lesions, the dorsal hair of all mice was shaved at week 0 using electric clippers and a depilatory cream (Fresh Hair Removal Cream, Tosowoong, Seoul, Korea). Symptoms were evaluated based on two parameters, the itching score and the severity score. The itching score was determined by reviewing video recordings to count the number of scratching bouts directed to the dorsal skin during a 15-minute observation period, as described by Tanaka and Matsuda^54^. The severity score was assessed using five criteria—dryness, excoriation, edema, erosion, and erythema—with each criterion rated on a scale from 0 to 3. The total AD score was calculated as the sum of itching and severity scores.

RNA was extracted from the intestinal and skin tissues of the test animals using a RNeasy Mini Kit (Qiagen, Hilden, Germany), and its concentration was measured using a Nano-Quant Spectrophotometer (Infinite 200; Tecan, Switzerland). Total RNA was reverse transcribed into cDNA using a PrimeScript First-Strand cDNA Synthesis Kit (Takara Bio, Shiga, Japan). RT-qPCR analysis was performed using a SYBR Green PCR kit (Qiagen) and a 7500 Fast Real-time PCR system. The fold change in gene expression was calculated using the ∆∆Ct method^55^.

### Serum cytokines and histological analysis

Blood samples were collected from the eyes of the test animals into 1.5 mL serum separation tubes, allowed to stand at 4 °C for 1 h, and centrifuged at 1,500 × *g* for 5 min to separate the serum. Serum levels of immunoglobulin E (IgE) and Th1/Th2-related cytokines (TNF-α, IFN-γ, IL-12, IL-4, IL-5, IL-13, IL-1β, TARC, and eotaxin) were measured using enzyme-linked immunosorbent assay (ELISA) kits (Abcam, Cambridge, UK; MyBioSource, San Diego, CA, USA), following the manufacturer’s protocols. Full-thickness skin samples were collected from the shaved dorsal region following euthanasia, rinsed with phosphate-buffered saline (PBS), and fixed in 4% paraformaldehyde for 16 h^2,8^. The tissues were embedded in paraffin, sectioned at 6 μm thickness, and stained with hematoxylin and eosin (H&E) for histological evaluation. Mast cells were visualized using toluidine blue staining (T3260, Sigma, USA)^56^. Stained tissues were analyzed using a panoramic viewer (SZX16; Olympus, Japan), and mast cell counts were determined in five randomly selected fields.

### Gut microbiota and Microbiome analysis, SCFA measurements

Genomic DNA was extracted from fecal samples using a FastDNA Spin kit (MP Biomedicals, Irvine, CA, USA) following the manufacturer’s protocol. The V3-V4 region of 16 S rRNA was amplified using the 347 F/803R primer set^2^, and sequencing libraries were prepared using the Illumina MiSeq system (Macrogen Inc., South Korea). Raw sequencing data were processed using the QIIME2 software (Ver. 1.9.1) with demultiplexing and noise removal using DADA2 (ver. 1.28.0)^57^. Chimeric sequences were filtered, and amplicon sequence variants (ASVs) were generated for diversity and taxonomy analyses. Taxonomy was assigned using the GreenGenes database (gg-13_8, 99%)^58^. Microbiota data were analyzed using R Studio (version 3.6.1) and diversity was assessed using the phyloseq R package (ver. 1.30.0). PERMANOVA (999 permutations) was performed using the Adonis function in the vegan R package. Taxonomic composition and heatmap visualizations were generated using the phyloseq and DESeq2 R packages (ver. 1.24.0). Quantification of SCFAs was performed by the National Instrumentation Center for Environmental Management (NICEM) at Seoul National University (Seoul, Korea). Fecal samples were homogenized in distilled water and centrifuged at 12,000 × g for 20 min at 4 °C. The resulting supernatant was used for analysis. SCFAs, including acetic acid, butyric acid, and propionic acid, were separated using high-performance liquid chromatography (Ultimate 3000, Thermo Dionex, Sunnyvale, CA, USA) equipped with an Aminex 87 H column (300 × 10 mm, Bio-Rad, Hercules, CA, USA)^2^. The mobile phase consisted of 0.01 N sulfuric acid (Fluka, La Jolla, CA, USA) delivered isocratically at a flow rate of 0.5 mL/min. Detection was carried out at 210 nm using a refractive index detector (RefractoMAX520, ERC, Tokyo, Japan).

### DNA extraction, WGS analysis, and gene annotation

Genomic DNA from LP5195 was extracted using a Genomic DNA Extraction Kit (Intron Biotechnology, Korea) following the manufacturer’s instructions, and analysis was performed as described by Lim et al.^9^. Bacterial cultures were grown in MRS broth at 37 °C under anaerobic conditions for 12 h to reach the exponential growth phase. Cells were harvested via centrifugation at 8,000 × g for 10 min at 4 °C, washed twice with sterile PBS, and lysed through incubation with 200 µL lysis buffer at 55 °C for 30 min, followed by the addition of Proteinase K (20 µL). DNA precipitation was achieved by adding binding buffer and isopropanol, and the DNA was pelleted through centrifugation at 10,000 × g for 5 min. The DNA pellet was washed with 70% ethanol and resuspended in elution buffer, then stored at − 20 °C. DNA quality and purity were assessed using a NanoDrop spectrophotometer (A260/A280 ratio of 1.8–2.0) and agarose gel electrophoresis. For WGS, genomic libraries were prepared using a 20 kb SMRTbell Libraries Prep Kit (Pacific Biosciences [PacBio], Menlo Park, CA, USA), and sequencing was performed on the PacBio RSII platform. Raw sequencing data were processed using the PacBio SMRT Analysis software (version 2.3.0) and assembled using a Hierarchical Genome Assembly Process (HGAP, version 3.0) to generate high-quality de novo genome assemblies. Gene annotation was conducted using RAST (https://rast.nmpdr.org/), the NCBI Prokaryotic Genomes Annotation Pipeline (www.ncbi.nlm.nih.gov/refseq/annotation_prok/), and PATRIC 3.5.43 online server for bacteria, with further refinement using eggNOG-mapper (ver. 2.0.1) to identify orthologous genes.

### Safety assessment in vitro

Antibiotic susceptibility was assessed by determining the minimum inhibitory concentrations (MICs) of ampicillin, tetracycline, chloramphenicol, erythromycin, vancomycin, clindamycin, and streptomycin, following the guidelines of the European Food Safety Authority (EFSA), using the broth microdilution method. Briefly, antibiotics were serially diluted two-fold in MRS broth in 96-well microplates, and 100 µL of the standardized bacterial suspension was added to each well. After incubation at 37 °C for 24 h under anaerobic conditions, MIC was defined as the lowest antibiotic concentration inhibiting visible growth, verified using OD measurements at 600 nm. The MIC values for LP5195 were compared to the EFSA-recommended cutoff values to assess susceptibility. Hemolytic activity was assessed to verify the safety of the strains for probiotic use. Bacterial strains were streaked onto Columbia blood agar plates supplemented with 5% defibrinated sheep blood and incubated anaerobically at 37 °C for 48 h. Hemolysis was classified as either positive or negative, with strains showing no lysis (γ-hemolysis) considered non-hemolytic and safe for probiotic use. Cell cytotoxicity was evaluated using MTT and lactate dehydrogenase (LDH) release assays. Caco-2 cells were obtained from the KCLB (30037.1, Seoul, Korea), seeded into a 96-well plate, and incubated with bacterial supernatants for 24 h. After incubation, 20 µL of MTT solution (5 mg/mL) was added to each well, followed by incubation for 4 h at 37 °C. The resulting formazan product was dissolved in 100 µL DMSO, and the absorbance was measured at 570 nm. Cytotoxicity was calculated by comparing the absorbance of the treated cells with that of the control cells. For the LDH release assay, 100 µL of culture medium was collected from each well and analyzed using a commercial LDH assay kit (LDH Assay Kit Cytotoxicity, ab65393, Abcam) according to the manufacturer’s instructions. LDH release was measured at 490 nm, and cytotoxicity was determined by comparing the LDH levels in treated cells with those in control cells. D-Lactate production was assessed using a D-lactate assay kit (D-lactate Assay Kit, ab65329, Abcam). Bacterial cultures grown in MRS broth at 37 °C for 48 h were centrifuged, and the supernatants were analyzed for D-lactate concentration by measuring absorbance at 450 nm. D-lactate levels were compared using a standard curve of known concentrations. To confirm the complete heat inactivation of LP5195, bacterial cultures were adjusted to 1 × 10⁹ CFU/mL, and 10 mL aliquots were transferred into 15 mL conical tubes for heat treatment. The samples were subjected to heat treatment at 70 °C, 80 °C, and 90 °C for various time intervals at each temperature. After each treatment, 20 µL of the sample was streaked onto MRS agar plates and incubated at 37 °C for 48 h. The presence or absence of colony formation on the agar was assessed to determine the survival of viable cells. Complete inactivation was defined as no colony formation observed after incubation.

### AD-alleviating effects of 5195-P in vitro

Rat basophilic leukemia (RBL-2H3) cells and human keratinocyte (HaCaT) cells were obtained from the Korea Cell Line Bank (KCLB, Seoul, Korea). RBL-2H3 cells were maintained in Dulbecco’s Modified Eagle’s Medium (DMEM; Gibco, Grand Island, NY, USA) supplemented with 10% fetal bovine serum (FBS), 100 U/mL penicillin, and 100 µg/mL streptomycin at 37 °C with 5% CO₂. Cells (2 × 10⁶ cells/mL) were seeded in 6-well plates and treated with 50 µM of LP5195-P or P-CNT for 30 min, followed by stimulation with 0.1 µg/mL of DNP-HSA. Supernatants were collected for the measurement of histamine release using an ELISA kit, and β-hexosaminidase activity was determined using p-nitrophenyl-N-acetyl-β-D-glucosaminide (p-NAG) with absorbance measured at 405 nm. Similarly, HaCaT cells were cultured under identical conditions and seeded at 5 × 10⁵ cells/mL in 6-well plates. Following treatment with 50 µM of LP5195-P or P-CNT for 24 h, supernatants were collected, and the levels of IL-4, IL-5, IL-13, TARC, and eotaxin were measured using ELISA kits following the manufacturer’s instructions.

### Statistical analysis

All Statistical analyses were performed using GraphPad Prism (version 9.1, GraphPad, San Diego, CA, USA). To evaluate relative differences, we determined statistically significant differences between groups by using the non-parametric Kruskal–Wallis test for microbial phyla and genera. One-way ANOVA was used for multiple group comparisons. Differences between groups were considered significant at *P* < 0.05.

## Data Availability

The datasets generated and/or analyzed during the current study are available in the National Center for Biotechnology Information (NCBI) repository. The whole genome sequence data have been deposited in the Sequence Read Archive (SRA) under BioProject accession number PRJNA1265405, BioSample SAMN48598991, and GenBank accession number JBPFSE000000000.These data can be accessed at: https://www.ncbi.nlm.nih.gov/bioproject/PRJNA1265405/.
