# Correction: Atopic dermatitis-alleviating effects of *lactiplantibacillus plantarum* LRCC5195 paraprobiotics through microbiome modulation and safety assessment via genomic characterization and in vitro analysis

**DOI:** 10.1038/s41598-025-22267-w

**Published:** 2025-09-29

**Authors:** Ahyoung Lim, Jihye Baek, Minju Seo, Ki-young Kim, Seonghan Kim, Woongkwon Kwak, Jaeho Lee, Jungki Kwak, Won-Joo Yoon, Wonyong Kim, Seokmin Yoon

**Affiliations:** 1 Lotte R&D Center, Seoul, 07594 Republic of Korea; 2 Department of Microbiology, Chung-Ang University College of Medicine, Seoul, 06974 Republic of Korea; 3 LuxBiome Co. Ltd, Seoul, 06974 Republic of Korea

Correction to: *Scientific Reports* 10.1038/s41598-025-16102-5, published online 20 August 2025

In the original version of this Article, Wonyong Kim was omitted as a corresponding author. Correspondence and requests for materials should also be addressed to: wonyong.kim@email.com.

The original Article has been corrected.

